# Nuclear factor E2 p45-related factor (NRF2) and peroxiredoxin 6 tissular expression as prognostic biomarkers for advanced HPV-negative squamous cell carcinoma of the oropharynx

**DOI:** 10.1016/j.tranon.2025.102595

**Published:** 2025-11-03

**Authors:** Pierre Philouze, Nazim Benzerdjeb, Céline Malésys, Anne-Sophie Wozny, Mamadou Soumboundou, Amandine Kobler, Alain C. Jung, Mickaël Burgy, Philippe Céruse, Gersende Alphonse, Claire Rodriguez-Lafrasse

**Affiliations:** a Cellular and Molecular Radiobiology, UMR CNRS5822/IP2I, Lyon-Sud Medical School, Univ Lyon 1, Lyon University, 69921 Oullins, France; b Department of OtoRhinoLaryngology Head and Neck Surgery, Croix-Rousse Hospital, Hospices Civils de Lyon, 69004 Lyon, France; c EA3738 CICLY, Univ Lyon 1, Lyon University, Department of Pathology, Lyon Sud Hospital, Hospices Civils de Lyon, 69310 Pierre-Bénite, France; d Biophysics Laboratory UFR Health, Senegal; e Department of Biochemistry and Molecular Biology, Lyon-Sud Hospital, Hospices Civils de Lyon, 69310 Pierre-Bénite, France; f Centre de ressources biologiques en cancérologie, Centre de Lutte contre le cancer Paul Strauss, France; g Cancerology Institut of Strasbourg Europe, Department of Medical Oncology, 67200 Strasbourg, France

**Keywords:** SCC, Oropharynx, NRF2, Peroxiredoxin 6, Prognosis biomarker

## Abstract

**Objectives:**

Non-HPV-induced squamous cell carcinomas (SCC) of the oropharynx have a poorer prognosis than HPV-induced cancers because they are more radio- and chemoresistant. Currently, no predictive biomarker exists for survival outcome in this type of tumor. NRF2 is a nuclear transcription factor that regulates the expression of cytoprotective genes, and peroxiredoxin 6 is one of the proteins regulated by NRF2. Both are potentially involved in the resistance to treatments as they play a central role in the response to oxidative stress.

**Material and Methods:**

In a retrospective cohort study of 53 patients with advanced HPV-negative squamous cell carcinomas of the oropharynx, we studied the correlation between clinical characteristics, response to treatment (surgery + radiotherapy), patient survival, and the expression of NRF2 and peroxiredoxin 6 in pre-treatment tumor biopsies. We prepared Tissue Micro Arrays (TMA) before performing immunohistochemistry analyses.

**Results:**

For a given patient, prior to any treatment, NRF2 and peroxiredoxin 6 are overexpressed in tumor tissue compared with healthy tissue. Moreover, patients with high NRF2 expression tended to have poorer progression-free survival (*p* = 0,09). Patients with high expression of peroxiredoxin 6 had significantly worse progression-free survival and overall survival (*p* = 0.015 and *p* < 0.01, respectively). The combination of high NRF2 and peroxiredoxin 6 expression was associated with even worse survival outcomes (*p* < 0.01and *p* = 0.01, respectively).

**Conclusion:**

Elevated expression levels of peroxiredoxin 6, either alone or in combination with NRF2, are poor prognostic indicators for patient survival.

## Highlights

• NRF2 and peroxiredoxin 6 are overexpressed in tumor tissue compared to healthy tissue prior to any treatment, suggesting their involvement in therapy resistance.
• High tumoral expression of NRF2 or peroxiredoxin 6 are negative prognostic factors on survival outcomes in non-HPV-induced oropharyngeal SCC patient.
• Associating peroxiredoxin 6 and NRF2 expression improves patient survival prediction.

## Introduction

Oropharyngeal squamous cell carcinomas are linked either to exposure to alcohol and tobacco exposure, or to HPV infection. Non-HPV-induced squamous cell carcinomas (SCC) of the oropharynx account for 40–60 % of oropharyngeal cancers and have a poorer prognosis because they are more radio- and chemo-resistant than HPV-induced cancers [1,2]. Radiotherapy is a standard and widely applied therapeutic modality for the management of these carcinomas [3]. However, no reliable tumor biomarker is currently available to predict radioresistance in HPV-negative tumors. The identification of such biomarkers would represent a critical step toward more personalized and effective treatment strategies.

The nuclear factor erythroid 2–related factor 2-Kelch-like ECH-associated protein 1 (NRF2-Keap1) pathway, which regulatesoxidative stress, may play a central role in their resistance to treatment [4].

Oxidative stress occurs in cells when there is an imbalance between the production and elimination of reactive oxygen species (ROS). The ROS-sensitive NRF2 transcription factor regulates the expression of antioxidant systems and anti-apoptotic proteins, conferring tumor cell protection against oxidative stress and apoptosis. Its transcriptional program includes a battery of genes, including those coding for enzymes involved in glutathione synthesis (glutamate cysteine ligase, and glutathione synthetase) and regeneration (glutathione reductase), and for the peroxiredoxin 6 (Prdx6) antioxidant enzyme. Keap1 is an NRF2-binding protein that regulates NRF2-dependent transcription by targeting NRF2 for proteasomal degradation. Under normal homeostatic conditions, Keap1, through its interaction with NRF2 in the cell cytoplasm, constitutively suppresses NRF2 activity.

Endogenous or exogenous oxidative stresses, such as irradiation, impair Keap1-mediated proteasomal degradation of NRF2. Oxidative stress leads to the dissociation of NRF2 from Keap1, resulting in nuclear translocation of NRF2 and transcriptional induction of its target genes [[5], [6], [7]]. NRF2 promotes cancer cell proliferation by reducing intracellular level of ROS. Some studies have shown that NRF gain of function resulting from the loss of interaction with Keap1, promotes tumor growth and confers chemoresistance in cancer cells [[8], [9], [10], [11]]. Other mechanisms involved in tumor progression are activated by NRF2 and have been explored in other tumors. For example, the epithelial-to-mesenchymal transition, which is involved in the metastatic process, is also regulated by NRF2 through decreasing the expression of E-cadherin [12]. In addition, NRF2 provides therapeutic resistance to standard treatments by increasing the detoxification capacity of cells and the activation of drug transporters [13]. NRF2 also represses inflammation by inhibiting the production of pro-inflammatory cytokines (IL6, IL1b) by macrophages [14]. In addition, the production of IL-11 driven by NRF2 has a tumorigenic effect. Recent studies suggest that the activation of PD-L1 by NRF2 contributes to immune escape in cancer [15,16].

The peroxiredoxins constitute a family of proteins acting as antioxidant enzymes involved in a variety of metabolic functions and pathways, including intracellular hydrogen peroxide (H_2_O_2_) reduction. These proteins help maintain intracellular ROS balance [17,18]. An imbalance in ROS is associated with tumor progression; therefore, the peroxiredoxin family is of particular interest in oncology. Peroxiredoxin proteins are dysregulated in many tumors including digestive and gynaecological cancers [[19], [20], [21], [22]]. Peroxiredoxin 6, regulated by NRF2, is a unique and multifunctional enzyme in the peroxiredoxin family, with glutathione peroxidase, calcium-independent phospholipase A₂, and lysophosphatidylcholine acyltransferase activities. Moreover, peroxiredoxin 6 is involved in the regulation of cell migration and is also associated with cancer progression [23,24]. Overall, the NRF2 pathway provides favorable conditions for cancer cells’ growth and survival.

In head and neck squamous cell carcinoma (HNSCC), NRF2 is overexpressed in 90 % of cases, and recent studies suggest that NRF2 pathway mutations may confer radioresistance in laryngeal squamous cell cancers [9,18]. Moreover, gain of function mutations in the NRF2 pathway are highly prevalent in HPV-negative oropharyngeal tumors [19].

Only a few studies have analyzed NRF2 expression in tumor samples from patients with HNSCCs. These studies identified NRF2 overexpression in tumor tissue compared with healthy tissue, as well as poorer overall and progression-free survival for patients with NRF2 overexpression [10,11,25,26]. However, these findings were limited either by the inclusion of heterogeneous anatomical sites or by the lack of consideration of patients’ HPV status. Investigations on tumor biopsies involving peroxiredoxin 6 are even rarer. Only one study showed that peroxiredoxin 6 is expressed at lower levels in buccal mucosa than in tumor tissue, highlighting the role of antioxidant enzymes in cancer development [27].

This study aimed to assess whether the expression of NRF2 and Peroxiredoxin 6 could serve as predictive biomarkers of tumor response to treatment (surgery combined with radiotherapy), as evaluated by the absence of tumor progression at 24 months. To achieve this, we included 53 tumor biopsies of patients with advanced HPV-negative oropharyngeal squamous cell carcinomas. We obtained these biopsies from surgical specimens before adjuvant radiotherapy.

## Materials and methods

### Population and tumor samples

We included 53 patients with advanced HPV-negative oropharyngeal SCC who underwent surgery followed by adjuvant radiotherapy (66 Gy in 33 fractions to the primary tumor and regional lymph nodes). We collected tumor samples from surgical biopsies before any treatment. All patients had given their consent for the storage of samples in the tumor biobank (Head and Neck Tumor Biobank, Centre Paul Strauss, Strasbourg, France). Authorization number: AC-2023–5681. We classified tumors using the 8th edition AJCC/UICC TNM staging system for HNSCC [28].

### Tissue microarray (TMA) analyses

We prepared TMAs from samples obtained from 53 patients recruited for squamous cell carcinoma of the oropharynx in the Head and Neck Surgery Department of Strasbourg University Hospital.For each patient, the TMAs were composed of 3 cores of 2-mm diameter of tumor tissue matched with 2 cores of 2-mm diameter of adjacent non-tumor tissue. To investigate the squamous cell carcinoma tissue of each patient, we sliced consecutive sections of each TMA block . The first section was transferred onto a glass slide to perform Hematoxylin & Eosin (H&E) counterstaining for cell identification by a pathologist specializing in head and neck neoplasms. The other sections were dedicated to immunohistochemistry with anti-NRF2 or anti-peroxiredoxin 6. The immunohistochemical analysis were performed with an automated immunostainer (BOND, Leica Microsystems, Wetzlar, DE). Briefly, formalin-fixed paraffin-embedded 4-μm-thick sections were first deparaffinized in xylene and rehydrated in ethanol. The endogenous peroxidase activity was blocked (Ventana Medical Systems, Tucson, Arizona, USA) before antigen retrieval was begun. The Ultra-C1 Cell Conditioning Solution (Ventana Medical Systems) was then used for antigen retrieval. Immunohistochemical staining was carried out on an automated immunostainer, with primary antibody: NRF2 (Sigma Aldrich, Tucson, Arizona, USA) and peroxiredoxin 6 (Abcam, Cambridge, UK). This was followed by the application of the avidin-biotin-peroxidase complex technique. Reactions were developed with diamino-3,3′-benzidine tetrahydrochloride substrate solution (Sigmafast; Sigma-Aldrich). The tissues were counterstained with hematoxylin. After performing the TMAs, the slides were automatically analyzed under a microscope using Metafer software. (MetaSystems GmbH, Altlußheim, DE). For each sample, staining intensity was given on a scale from 0 to 10 000 AU We then calculated the average staining intensity for each patient across the three tumor tissue cores and two healthy tissue cores.We applied this methodology for NRF2 and peroxiredoxin 6.

### Statistics

We used Student’s *t*-test to compare the expression levels of NRF2 and Peroxiredoxin 6 between healthy and tumor tissues, as well as to assess associations between biomarker expression and clinical characteristics.

We investigated the correlation between NRF2 and peroxiredoxin 6 expression in tumor biopsies using Pearson’s correlation test. For each biomarker, we fitted a logistic regression model for responder vs. non-responder patients, using categorized biomarker scores as the independent variable.. Receiver operating characteristic (ROC) curves based on the logistic regression model were plotted. Sensitivity, specificity, and areas under the curve (AUC) of the ROC curve based on the trapezoidal rule were calculated. An optimal cut-off was calculated using Youden's method for each biomarker. To define this cut-off as objectively as possible, the five patients with the fastest local progression and the five patients with the longest response time were considered for the calculation. We applied these cut-offs to the entire population to classify patients into NRF2-high, NRF2-low, PEROX-high, PEROX-low, NRF2/PEROX-high, and NRF2/PEROX-low groups.We compared progression-free survival (PFS) and overall survival (OS) among these subgroups using the log-rank test and Kaplan-Meier curves.Statistical analyses were performed using GraphPad® software (GraphPad Prism 8.4.2, GraphPad Software, San Diego, CA, USA).

## Results

### Patients characteristics

All 53 patients underwent surgery and adjuvant radiotherapy and all tumors were p16 negative. We obtained tumor samples based on the following criteria: oropharyngeal location, HPV-negative status, prior surgical treatment followed by adjuvant radiotherapy, and classification as responders (no tumor progression or events within the first 24 months of follow-up) or non-responders to radiotherapy.Table 1 summarizes patient characteristics.The 2 groups (26 responders and 27 non-responders) were well balanced.

**Table 1:** Clinico-pathological characteristics of patients. T, N and M scores were taken from TNM staging according to the AJCC/UICC eighth edition for HNSCC [28].

Characteristics	Values n ( %)
Age (mean ± SD)	57 ± 8
Sex	
Male	50 (94 %)
Female	3 (6 %)
Tumor location	
Tonsil	16 (30 %)
Base of tongue	17 (32 %)
Posterior pharyngeal wall	2 (4 %)
Palatoglossal arch	11 (21 %)
Soft palate	3 (6 %)
Adjacent locations	4 (7 %)
HPV status (p16 negative)	53 (100 %)
Tobacco	43 (81 %)
Alcohol	53 (100 %)
T stage	
T1	1 (2 %)
T2	24 (45 %)
T3	21 (40 %)
T4	7 (13 %)
N stage	
N0	4 (7 %)
N1	14 (27 %)
N2a	3 (6 %)
N2b	18 (35 %)
N2c	10 (18 %)
N3	4 (7 %)
M stage	
M0	53 (100 %)
M1	0 (0 %)
Treatment	
Surgery	53 (100 %)
Adjuvant radiotherapy	53 (100 %)
Responders/Non-responders at 24 months	
Responders	26 (49 %)
Non-responders	27 (51 %)

### Expressions of NRF2 and peroxiredoxin 6 in tumor versus normal tissue

**Fig. 1:**
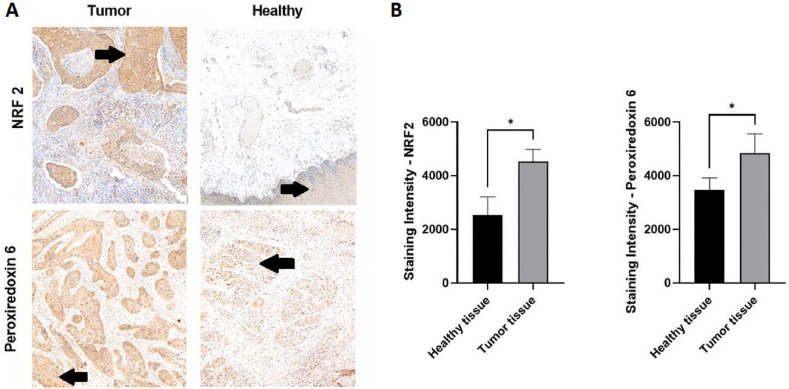
Comparison of NRF2 and peroxiredoxin 6 expressions between healthy and tumor tissues prior to treatment. A. NRF2 and peroxiredoxin 6 expression in a representative biopsy containing both tumor and adjacent healthy tissue. Circled areas are the analyzed areas. B. Quantifications of NRF2 and peroxiredoxin 6 expressions between tumor and healthy tissue from 6 representative biopsies (Student's *t*-test), *p* < 0.05.

We compared NRF2 and peroxiredoxin 6 expression between healthy and tumor tissues using biopsies from six representative patients. After identifying areas of interest in tumor and healthy tissues (Fig. 1A), we quantified NRF2 and peroxiredoxin 6 expression by measuring labeling intensity (Fig. 1B),revealing significant differences between tumor and healthy tissues. Both NRF2 and peroxiredoxin 6 were significantly overexpressed in tumor tissue compared with healthy tissue (*p* = 0.02 and *p* = 0.02, respectively).

### Correlation between NRF2 and peroxiredoxin 6 expression and clinical characteristics

**Fig. 2:**
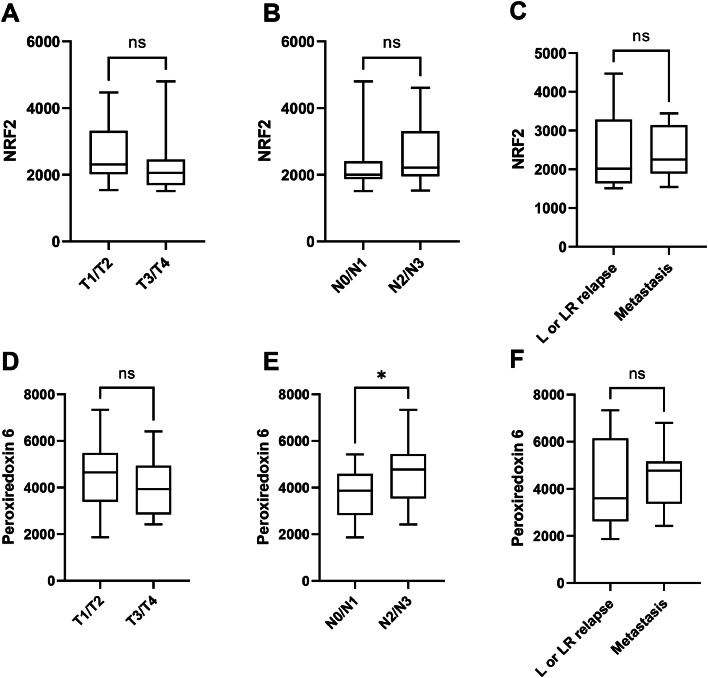
Correlations between NRF2 or peroxiredoxin 6 expression and clinical characteristics for the entire population (*n* = 53). [A] Comparison of the tumor status T1T2 versus T3T4 for NRF2, [B] Comparison of lymph node status N0N1 versus N2N3 for NRF2, [C] Comparison of local or locoregional relapse versus metastasis for NRF2, [D] Comparison of the tumor status T1T2 versus T3T4 for peroxiredoxin6, [E] Comparison of lymph node status N0N1 versus N2N3 for peroxiredoxin 6, |F] Comparison of local (L) or locoregional (LR) relapse versus metastasis for peroxiredoxin 6.

We investigated whether NRF2 or peroxiredoxin 6 expression could predict clinical characteristics such as tumor status, lymph node status, or recurrence type (Fig. 2)Among all these analyses, no significant correlation was found between NRF2 or peroxiredoxin 6 expression and either tumor status or recurrence type. However, we identified a significant association between peroxiredoxin 6 expression and lymph node status (*P* < 0.05) (Fig. 2E).

### Determination of NRF2 and peroxiredoxin 6 expression thresholds to identify responder and non-responder patients

**Fig. 3:**
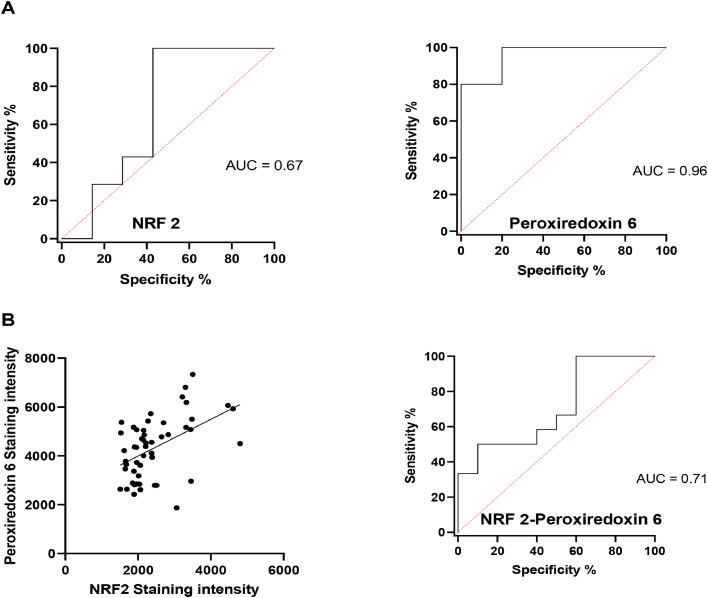
A. Analysis of the discriminatory capacity of NRF2 and peroxiredoxin 6 with the AUROC statistical analysis. For each marker an area under the curve was defined: AUC NRF2: 0.67, AUC peroxiredoxin 6: 0.96, AUC NRF2/peroxiredoxin 6: 0.71. B. Correlation between NRF2 and Peroxiredoxin 6 expression: Pearson correlation test. r² = 0.5 and *p* < 0.001 and AUROC statistical analysis for the combination of the two markers.

From the previous results, it appeared important to define whether the expression of NRF2 or peroxiredoxin 6 could be a relevant predictive biomarker for treatment response of patients. Based on their expression level in tumor tissue, a threshold that determines whether a patient responds to treatment or not was defined using AUROC (Area Under the Receiver Characteristic Curve) statistical analysis. The cut-offs were 1902 arbitrary units (AU) for NRF2 and 5923 AU for peroxiredoxin 6.Patients with a score above this cut-off were considered as non-responders to treatment, whereas patients with a lower score were considered as responders. The area under the curve (AUC), which can be used as a criterion to measure the discriminatory ability of the marker, is presented in Fig. 3A. With an AUC of 0.67 and 0.96 for respectively NFR2 and peroxiredoxin 6, these two markers can be considered reliable predictive markers. As both markers, taken independently, have a good AUC, it was important to define if combining the markers could improve the discrimination between responders and non-responders. First, the correlation between the expression of peroxiredoxin 6 and NRF2 was calculated (Fig. 3B). A Pearson correlation test was performed and showed a significative correlation with a r² of 0.5 and *p* < 0.001. The AUC calculation was therefore carried out and shows that the combination of both markers gives an AUC of 0.71.

### Expression of NRF2 and peroxiredoxin 6, alone or in combination, as a prognostic biomarker of progression-free and overall survival

**Fig. 4:**
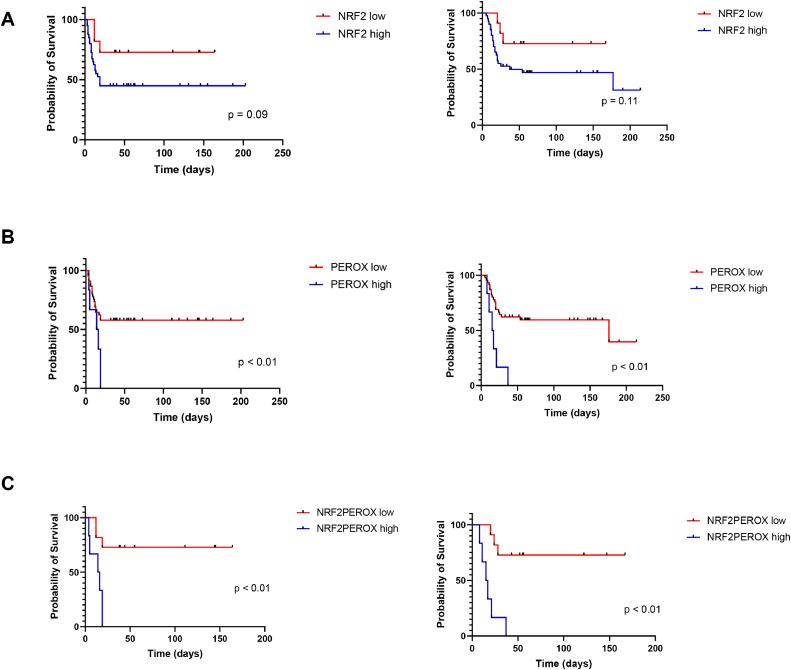
Progression-free survival (PFS) and overall survival (OS) of the NRF2 high and NRF2 low groups [A]; the PEROX high and PEROX low groups [B] and the NRF2PEROX high and NRF2PEROX low groups [C].

Based on the AUROC-determined cut-offs, we stratified patients into low- or high-expression groups for NRF2 and peroxiredoxin 6.Firstly, the PFS and OS of the patient with a high NRF2 expression were compared to the group with a lower expression (Fig. 4A). Regarding PFS, a tendency for better survival was observed for the NRF2-low group (*p* = 0.09), whereas no such trend was observed for OS (*p* = 0.1). In contrast, high expression of peroxiredoxin 6 was significantly associated with poorer patient outcomes, both in terms of progression-free survival (*p* = 0.015) and overall survival (*p* < 0.01).

Among patients with concordant expression levels of both NRF2 and peroxiredoxin 6 (i.e., both high or both low; *n* = 17), we identified a stronger correlation with clinical outcomes.High co-expression of the two markers was significantly associated with reduced progression-free survival (*p* < 0.01) and overall survival (*p* < 0.01).

## Discussion

This study aimed to determine whether immunohistochemical analysis of the Keap1/NRF2 pathway—by assessing basal NRF2 and peroxiredoxin 6 expression in tumor biopsies—could serve as a prognostic biomarker for treatment response and survival in patients with non-HPV-induced oropharyngeal SCC.In squamous cell carcinomas of the oropharynx, apart from HPV status, no reliable prognostic biomarkers are currently available. When patients present with a tumor not induced by HPV, and often associated with alcohol and tobacco use, the prognosis is poor with a survival of around 50 % at 5 years [1]. Identifying predictive biomarkers for treatment response and survival is therefore critical.This effort would aid in identifying patients who may require more intensive treatment. The search for predictive markers of treatment response in tumor biopsies at the time of diagnosis appears to be a promising and feasible strategy, particularly because immunohistochemistry markers are routinely used in all pathology laboratories. In this work, it was important to identify biomarkers that are easy to quantify routinely and highly reproducible, in order to enable timely clinical management of patients, as these tumors progress rapidly and early treatment improves outcomes [29]. Radiotherapy is a standard treatment modality for oropharyngeal tumors, used either alone, in combination with chemotherapy, or as an adjuvant to surgery [3]. In this context, we focused on NRF2 and the antioxidant protein peroxiredoxin 6, both of which are key regulators of the cellular response to oxidative stress. Exogenous oxidative stressors, such as ionizing radiation, disrupt Keap1-mediated proteasomal degradation of NRF2. This results in the dissociation of NRF2 from Keap1, its subsequent nuclear translocation, and the transcriptional activation of downstream cytoprotective genes [[5], [6], [7]]. By reducing intracellular ROS levels, NRF2 fosters cancer cell survival and proliferation, thereby contributing to therapy resistance.

Beyond radiation, toxicological studies conducted on non-oncological models show that oxidative stress is a key factor in cellular damage. Indeed, exogenous factors, including arsenic, fluoride, and microplastics, can alter cellular redox status by promoting ROS overproduction and impairing antioxidant defenses, including Nrf2-related pathways [[30], [31], [32], [33]]. Taken together, these data reinforce the idea that dysregulation of the NRF2 antioxidant system could play a crucial role not only in environmental toxicity, and possibly in carcinogenesis but also in tumor resistance to treatment.

Thus, we have investigated if a relationship between tumor expression of NRF2, peroxiredoxin 6, and survival in a cohort of patients with advanced non-HPV induced oropharyngeal squamous cell carcinomas treated with surgery followed by adjuvant radiation therapy exists.

To date, some studies confirm the important role of NRF2 in tumor progression in HNSCC [10,11]. In contrast to the physiological regulation of NRF2, there is evidence of increased basal NRF2 activation in tumors [10,25]. Indeed, some mutations that disrupt the NRF2-Keap1 interaction to stabilize NRF2 and thus increase transcription of NRF2 target genes have been identified. This leads to improved ROS detoxification and therefore resistance to treatments such as radiotherapy. Recent evidence suggests that NRF2 pathway mutations promote radioresistance, particularly in laryngeal SCC [11]. In addition, another study demonstrated that NRF2 enhances the expression of the antioxidant enzyme GPX2, which plays a pivotal role in maintaining cancer stem cell populations through the upregulation of NOTCH3, a key driver of tumor progression [9].NRF2 also has other pro-tumorigenic functions. NRF2 promotes metastasis by regulating epithelial-mesenchymal transition and modulating cytokine production—suppressing pro-inflammatory cytokines (e.g., IL-6, IL-1β) while inducing tumor-promoting ones like IL-11. It also enhances immune evasion by upregulating PD-L1 [15]. Overall, the NRF2 pathway creates favorable conditions for cancer cell growth and survival.In our study, we demonstrated a significant difference in basal NRF2 expression between healthy and tumor tissues. Moreover, concerning patient survival, we have observed that a high expression of NRF2 is associated with a tendency toward poorer survival.

The role of peroxiredoxin 6, an antioxidant enzyme regulated by NRF2, as a prognostic marker was also investigated. Previously, its implication in tumor resistance has been analyzed in other localizations such as lung, breast, and ovarian tumors. As an example, overexpression of peroxiredoxin 6 promotes the growth of lung cancer cells by upregulating AP-1 and JNK, thus increasing some peroxidases and phospholipase A2 activities [34]. Regarding ovarian cancer, overexpression of peroxiredoxin 6 leads to the inhibition of cisplatin-induced apoptosis [35]. In human breast cancer cell lines, peroxiredoxin 6 overexpression leads to a more invasive phenotype, increased metastatic potential, and drug resistance [36,37]. In tumor biopsies, studies of other peroxiredoxins, such as peroxiredoxin 5 or peroxiredoxin 1 have demonstrated that their expression before any treatment is able to predict clinical outcomes [38,39]. Finally, in our study, we confirmed the data of Huang *et al*. who demonstrated that, in HNSCC, peroxiredoxin 6 is more highly expressed in oral cavity squamous cell carcinomas than in healthy mucosa [27]. Moreover, as for NRF2, a high expression of peroxiredoxin 6 is significantly correlated with poor survival. Furthermore, analysis of the results according to clinical characteristics shows that high expression of peroxiredoxin 6 is related to patients with advanced lymph nodes.

While our study provides preliminary evidence of the association between NRF2/PRDX6 and patient prognosis, the limited size of our cohort (*n* = 53) and the small subgroup used for certain comparisons (*n* = 6) require cautious interpretation of the results. Prospective studies with larger cohorts are needed to validate these observations. Our results suggest that PRDX6 expression, alone or in combination with NRF2, could serve as a potential predictive marker for survival in patients with HPV-negative oropharyngeal SCC. However, the borderline statistical significance of NRF2 (*p* = 0.09) and its moderate AUC (0.67) highlight the need to validate these findings in independent cohorts before considering them clinically reliable markers. The expression thresholds for NRF2 (1902 AU) and PRDX6 (5923 AU) were determined using Youden’s method, based on ROC curves. However, these thresholds were not biologically calibrated nor validated in other laboratories, which represents a limitation for their clinical application. Standardization of immunohistochemistry protocols and external validation are necessary to ensure the reproducibility of these results.

The proposed mechanisms explaining the role of NRF2 and PRDX6 in therapeutic resistance are partly based on data from other cancer types (lung, breast, ovarian). While these studies provide a solid biological rationale, functional investigations specific to oropharyngeal SCC are needed to confirm these hypotheses. While a Cox model was not included in this study, such an analysis would be relevant to adjust for confounding factors (T/N stage, age, smoking) and strengthen the robustness of the observed associations. This will be the subject of future work.

To our knowledge, our study is the first to show that peroxiredoxin 6 overexpression significantly impacts patient survival.We also showed that patients with lymph node at diagnosis had higher peroxiredoxin 6 expression . This could partly explain the poorer survival, as it is widely accepted that lymph node involvement is associated with a worse prognosis. Finally, combining the analysis of NRF2 and peroxiredoxin 6 expression slightly improved survival prediction.

## Conclusion

Our findings uncover a previously unrecognized connection between NRF2 and PRDX6 signaling in OPSCC, which is associated with adverse clinical outcomes. Targeting this axis may represent a promising strategy to overcome oxidative stress–driven therapy resistance. Further validation in larger, independent cohorts, together with mechanistic and functional studies, will be essential to confirm its clinical relevance and therapeutic potential.

## Funding

This research was funded by Ligue of Haute-Savoie, Nuovo Soldati Fondation and LABEX PRIMES (ANR-11-LABX-0063) of Lyon University, within the program “Investissements d’Avenir” (ANR-11-IDEX-0007) operated by the French National Research Agency (ANR).

## Informed consent statement

Informed consent was obtained from all subjects involved in the study. Tumor bank authorization number AC-2023–5681.

## CRediT authorship contribution statement

**Pierre Philouze:** Writing – review & editing, Writing – original draft, Visualization, Validation, Supervision, Software, Resources, Project administration, Methodology, Investigation, Funding acquisition, Formal analysis, Data curation, Conceptualization. **Nazim Benzerdjeb:** Writing – review & editing, Visualization, Validation, Methodology, Data curation. **Céline Malésys:** Writing – review & editing, Investigation, Data curation. **Anne-Sophie Wozny:** Writing – review & editing, Visualization, Validation, Methodology, Formal analysis. **Mamadou Soumboundou:** Writing – review & editing, Investigation, Data curation. **Amandine Kobler:** Writing – review & editing, Investigation, Data curation. **Alain C. Jung:** Writing – review & editing, Validation, Investigation, Data curation. **Mickaël Burgy:** Writing – review & editing, Methodology, Investigation, Data curation. **Philippe Céruse:** Writing – review & editing, Supervision, Conceptualization. **Gersende Alphonse:** Writing – review & editing, Visualization, Validation, Methodology, Formal analysis, Data curation. **Claire Rodriguez-Lafrasse:** Writing – review & editing, Writing – original draft, Visualization, Validation, Supervision, Resources, Methodology, Investigation, Funding acquisition, Formal analysis, Conceptualization.

## Declaration of competing interest

The authors declare no conflict of interest

## Appendix Supplementary materials

Image, application 1
