# Supplementary material for: Nuclear factor E2 p45-related factor (NRF2) and peroxiredoxin 6 tissular expression as prognostic biomarkers for advanced HPV-negative squamous cell carcinoma of the oropharynx
Source: Transl Oncol. 2025 Nov 3;63:102595. doi: 10.1016/j.tranon.2025.102595 (PMC12630086; doi:10.1016/j.tranon.2025.102595)
Supplement: Supplementary file 1 [file mmc1.pdf]

Oral Oncology
Author Form

All manuscripts submitted to *Oral Oncology* must be accompanied by this form. Please scan the form and transmit it to the Editorial Office via EES with the manuscript. If you are unable to do this, please contact the Editorial Office at ooncology@elsevier.com to organise an alternative way of sending the form to the *Oral Oncology*.

Title of Manuscript:

Nuclear factor E2 p45-related factor (NRF2) and peroxiredoxin 6 tissular expression as prognostic biomarkers for advanced HPV-negative squamous cell carcinoma of the oropharynx

**Contribution**	**Author(s)**
Study concepts:	PP CRL PC
Study design:	PP PC CRL
Data acquisition:	PP CM NB MS MB AJ ak
Quality control of data and algorithms:	NB CRL ASW PP
Data analysis and interpretation:	PP CRL NB GA
Statistical analysis:	PP NB GA
Manuscript preparation:	PP GA CRL
Manuscript editing:	PP ASW CRL GA
Manuscript review:	ALL AUTHORS

Ethical Approval for Research: No / Yes / N.A. (Yes circled)

External Funding: No / Yes
Source of Funding: Ligue of haute savoie, Nuovo-Soldati Foundaton, ANR-11-LABX-0063
Name of Principal Investigator: Pierre Philouze / Claire Rodriguez-Lafrasse
(If funded, please include a statement as to the role of the study sponsor at end of manuscript under a heading 'Role of the Funding Source')

Possible Conflict of Interest: No / Yes (No circled)
(Please ensure that a 'Conflict of Interest' statement is included in your manuscript)

Number of Tables: 1 **Number of Figures:** 4

Name and Title of Corresponding Author: Pierre PHILOUZE , MD PhD

Address: 103 grande rue de la Croix-Rousse
Address: ……………………
Postcode and country: 69006 Lyon, FRANCE
Tel No: +33426732774
Fax No: ……………………
Email: pierre.philouze@chu-lyon.fr

"I confirm that all the authors have made a significant contribution to this manuscript, have seen and approved the final manuscript, and have agreed to its submission to the *Oral Oncology*".

Signed (corresponding author): ……………………
Date: 07/07/25
